# Pulmonary alveolar microlithiasis with calcified pleural plaques

**DOI:** 10.4103/0970-2113.71967

**Published:** 2010

**Authors:** Balbir Malhotra, Raghu Sabharwal, Mandeep Singh, Amarjeet Singh

**Affiliations:** *Department of Chest and TB, Government Medical College Amritsar, India*; 1 *Department of Pathology, Government Medical College Amritsar, India*

**Keywords:** Microlithiasis, pulmonary, alveolar

## Abstract

Pulmonary alveolar microlithiasis (PAM) is a rare disease. Herein we report a case of pulmonary alveolar microlithiasis who was suspected to have the disease on chest X-ray and was confirmed on high resolution CT and transbronchial lung biopsy. These investigations showed characteristic features of pulmonary alveolar microlithiasis with diffuse interstitial pulmonary fibrosis.

## INTRODUCTION

Pulmonary alveolar microlithiasis (PAM) is a rare, chronic lung disease characterized by the presence of round little bodies containing concentric calcareous lamellas in pulmonary alveolus. There is paucity of symptoms in contrast to the imaging findings. The radiographic appearance of the disease is pathognomonic, chest radiographs show diffuse micronodular shadows. Earlier, the diagnosis was primarily made at autopsy, whereas nowadays diagnosis is made by transbronchial biopsy and bronchoalveolar lavage. Etiology and pathogenesis of pulmonary alveolar microlithiasis is not known. There are certain hypothesis for the etiology and pathogenesis of pulmonary alveolar microlithiasis but none of them are satisfactory. The incidence is similar in all continents, in both sexes and it is higher in age between 20 and 50 years. Familial incidence has also been reported by some authors. There is no known specific treatment to date. We herein report a case of pulmonary alveolar microlithiasis who was suspected to have this on chest X-ray and was confirmed on high resolution CT and transbronchial lung biopsy.

## CASE REPORT

**Figure 1:**
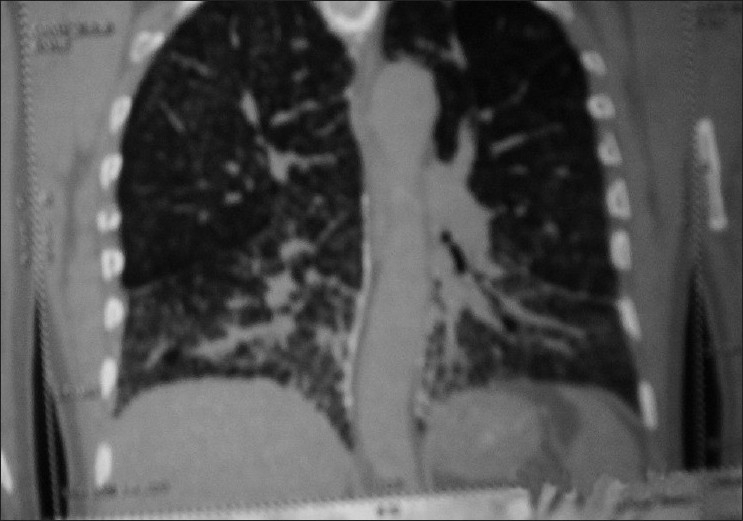
CT scan revealing diffuse bilateral calcified fine nodular pattern with extensive septal thickening

**Figure 2:**
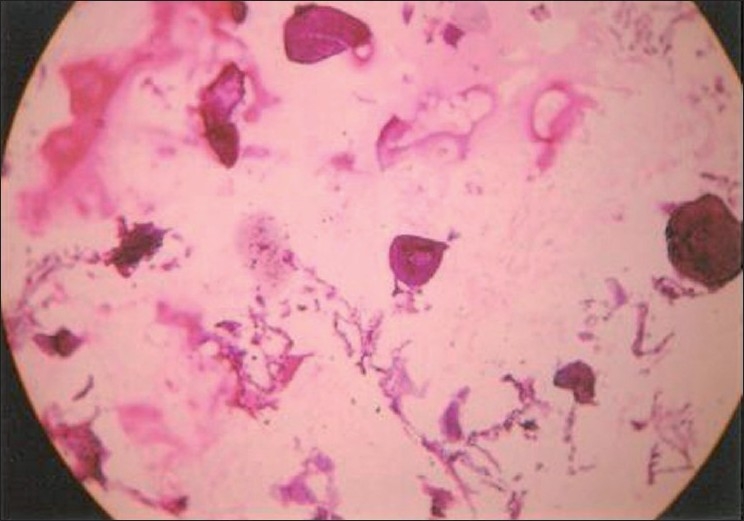
Bronchoscopic biopsy demonstrating typical intraalveolar and intraparenchymal concentric lamellar bodies

A 50-year-oldmale, non smoker, farmer by occupation reported in our hospital with breathlessness on exertion for the last 25 years which was almost the same since its onset. No history of wheeze, cough, fever, chest pain, anorexia, weight loss or recent change in voice was there. Vitals were within normal range and no anomaly was detected on detailed examination of the chest and other systems. The hemoglobin, total and differential leucocyte counts were within normal limits. Renal and liver function tests were also within normal limits. Results of urinalysis, skin tests for tuberculosis, and studies for acid-fast bacilli were negative. Serum calcium levels were below normal (6.4 mg/dl), probably due to excessive calcium deposition in the lung tissue (dystrophic calcification) leading to decrease in serum calcium levels. Chest radiograph revealed bilateral micronodular sand-like opacities more so in middle and lower zones. His family members were X rayed, but none of the family members showed any abnormal radiological feature. Pulmonary function tests revealed typical features of a restrictive defect with a reduced vital capacity of 1.88L(55% predicted), and a forced expiratory volume in 1 s/forced vital capacity (FEV1/FVC) of 113%. CT scan [Figure 1] revealed thickening of the interlobular, intralobular and septal interstitium with areas of fibrosis involving both the lung fields, more so at the bases. Also there was evidence of multiple tiny calcified nodular opacities within the alveoli scattered in both the lung fields and multiple calcified plaques were seen along the costal pleura bilaterally, predominantly in the lower lobes. These features were consistent with the diagnosis of pulmonary alveolar microlithiasis. Transbronchial lung biopsy [Figure 2] was done which showed the infiltration of interstitium with chronic inflammatory cells, mainly the lymphocytes, plasma cells and monocytes along with areas of fibrosis. Also calcified spherules were seen both in alveolar spaces and in interstitium thus proving the diagnosis of pulmonary alveolar microlithiasis.

## DISCUSSION

Pulmonary alveolar microlithiasis is a rare entity. Worldwide literature on pulmonary alveolar microlithiasis upto the end of 2001 reported 424 cases. The highest number of cases has been reported in Europe, followed by Asia. In India only 22 cases were reported up to 2001. Malpighi, was the first in the world to give a concise and precise macroscopic description of the disease, in 1686: ’In vesciculis pulmonum innumeri lapilli sunt’.[1] Much later, in 1918, the Norwegian Harbitz provided an accurate autopsy and radiological description of a second case[2] but it was Puhr who in 1933 named this disease as Micralithasis alveolaris pulmonum.[3] The definitive work on the subject was published by Sosman *et al* on the basis of 26 cases.[4] The disease has been recognized in a wide age range and there is no obvious sex difference and the disease has been reported from almost all parts of the world. In India the first case was reported by Viswanathan in 1962[5] and this institute has the privilege of reporting 4 cases of pulmonary alveolar microlithiasis till now (excluding this one).[6,7] Mikhaylov was the first to report the familial occurrence of pulmonary alveolar microlithiasis[8] and was supported by Sosman *et al*.[9] In over half the reported cases, a familial incidence has been demonstrated almost invariably among siblings. In our case the chest X–ray of the sibling of the patient was normal and none of the family member was having any H/o of respiratory illness. The etiology of the disease is still unknown. A genetic factor has been postulated because of familial occurrence. Sosman *et al* and Caffrey and Altman[10] have suggested the possibility of congenital error of metabolism at the level of alveolar surface membrane, possibly an enzymatic fault, resulting in the precipitation of calcium in the presence of undue alkalinity. Sharp and Danino believe that microliths develop in both lungs following single inflammatory or vascular episode, causing a generalized alveolar exudate based on the general uniformity of size and appearance of microliths.[11] Baar and Ferguson *et al*, believe that the condition is the result of an inflammatory process, probably hyper immune to one or more likely a variety of irritants.[12] Badger *et al*[13] attribute the condition to dysfunction of the parathyroid or other endocrine organs or to an abnormality of calcium metabolism. Tao[14] has postulated that the microliths are originally formed over the Curshmann’s spirals in the smaller bronchi or bronchioles rather than in the alveoli as generally speculated as the nuclei of microliths appear to be the loops (Coiled parts) of Curshmann’s spirals. The diagnosis of pulmonary alveolar microlithiasis is based on two findings

1. Characteristic radiological appearance which is bilateral sand-like micro nodules of calcific density, usually most marked in the middle and lower zones with relative sparing of the apices.
2. Striking clinico-radiological dissociation. Most of the cases are asymptomatic at the time of diagnosis, when the diagnosis is made by routine X-Ray of chest done for some other purpose. Although the radiological picture is diagnostic, many cases are mistaken for miliary tuberculosis, silicosis, berylliosis, sarcoidosis, hemosiderosis, fungal infections and carcinomatosis. Most patients remain symptom-free for many years despite extensive radiological changes. These two findings are very characteristic in our case. The chest X-ray is showing typical sand-like micronodulation diffusely involving both the lungs. The patient does not have much of the symptoms as compared to the involvement of the lungs. Although pulmonary function tests may initially yield normal results, more severely affected patients demonstrate a restrictive pattern along with impaired diffusing capacity. Our case too showed a restrictive pattern on lung function studies. HRCT shows a unique and characteristic calcified reticular pattern and thickening of the interlobular septa of the lung parenchyma, with predominant basal and peripheral lung distribution. The presence of calcified interlobular septal thickening on HRCT is due to the high concentration of microliths in the periphery of the secondary lobule rather than calcification of the interlobular septa. In some cases reticulonodular changes of the interlobular septa and intralobular interstitial lines associated with subpleural air cysts and paraseptal emphysema are evident, responsible for the paracostal “black line”, described in almost all cases in the literature. The disease diffusely involves all lung fields with a predominant distribution in the basal and peripheral lungs.[15,16] Some authors believe that the HRCT findings are pathognomonic for pulmonary alveolar microlithiasis and the open biopsy may be avoided in the presence of these characteristic findings. The pathological features are mostly limited to the lungs and the microliths are almost invariably intralveolar. The microscopic picture shows alveolar spaces containing typical laminated calcific microliths with fibrosis and thickening of the alveolar walls. Microliths have also been reported in the alveolar walls and alveolar septa. In our case microliths were present in both the alveoli and the interstitium. BAL and transbronchial biopsy respectively show the characteristic calcospherites in the recovered BAL fluid and in the alveolar spaces.[17] These two techniques have largely replaced open lung biopsies in providing histological confirmation.[18]

## CONCLUSION

There is currently no effective medical therapy, and affected individuals may progress to end-stage lung disease. The radiological picture may mimic miliary tuberculosis and the physician is often tempted to treat it with antitubercular drugs, thus adding on to the misery of patient. As more and more cases are reported there is a need to gain insight into its etiology and pathogenesis, which are still unknown. It will only be possible to develop effective treatment of the disease after its etiology has been fully established.
